# A Compilation of Study Models for Dental Pulp Regeneration

**DOI:** 10.3390/ijms232214361

**Published:** 2022-11-18

**Authors:** Ella Ohlsson, Kerstin M. Galler, Matthias Widbiller

**Affiliations:** 1 Department of Operative Dentistry and Periodontology, Friedrich-Alexander-University Erlangen-Nuernberg, D-91054 Erlangen, Germany; 2 Department of Conservative Dentistry and Periodontology, University Hospital Regensburg, D-93053 Regensburg, Germany

**Keywords:** regenerative endodontics, study model, dental pulp, regeneration, tissue engineering, cell culture techniques, animal models, translational research

## Abstract

Efforts to heal damaged pulp tissue through tissue engineering have produced positive results in pilot trials. However, the differentiation between real regeneration and mere repair is not possible through clinical measures. Therefore, preclinical study models are still of great importance, both to gain insights into treatment outcomes on tissue and cell levels and to develop further concepts for dental pulp regeneration. This review aims at compiling information about different in vitro and in vivo ectopic, semiorthotopic, and orthotopic models. In this context, the differences between monolayer and three-dimensional cell cultures are discussed, a semiorthotopic transplantation model is introduced as an in vivo model for dental pulp regeneration, and finally, different animal models used for in vivo orthotopic investigations are presented.

## 1. Introduction

The dental pulp has important functions, and its loss can have serious consequences. A root-filled tooth may remain in the oral cavity without pulp, but it lacks the ability to react to sensory stimuli, issue an immune response, or form reparative dentin [1]. Additionally, remaining hard tissue is weakened, and as a result, root fractures occur more frequently than in vital teeth [2]. If immature teeth are affected, root development comes to a halt, leaving thin dentin walls and an open apex behind, which complicates further therapies [3,4]. To overcome the biological and mechanical drawbacks of traditional endodontic treatment, research focused on pulp regeneration has gained interest over the last years. Several approaches in the realm of endodontic tissue engineering are being explored, which can be categorized into primarily cell-free methods, where resident stem cells re-populate the root canal, and cell-based approaches, where cells are introduced by transplantation [5]. In both approaches, the three pillars of classical tissue engineering, i.e., stem cells, signaling molecules, and a scaffold material, are present [6].

Interestingly, endodontic tissue engineering has already been translated into randomized clinical trials. Patients with irreversible pulpitis have been treated with transplantation of autologous [7] or allogenic [8] mesenchymal stem cells into root canals. In these studies, all teeth that received treatment through tissue engineering have survived after 12 months, and even positive responses to sensitivity testing were evident in a considerable number of cases. Further observations, such as radiographic reduction in apical lesion size and root lengthening and thickening, demonstrated clinical success [9]. However, the question remains whether this success is associated with biological regeneration of the pulp or a repair process. Teeth that have undergone regenerative procedures and are later extracted for other reasons often show proof of repair by ectopically formed tissues instead of restitutio ad integrum [5]. Strengthening the tooth root by the apposition of any hard tissue may be of clinical value and could contribute to increased mechanical resistance, but functional issues, e.g., adequate biological response of the dental pulp to external stimuli, remain unresolved [3,10]. In this context, histological examination is the only way to determine the exact nature of newly generated tissues. However, this is, of course, impossible in a systematic way in clinical studies. For this reason, preclinical study models are still indispensable for the development of new pulp regeneration procedures and for the biological evaluation of outcomes.

**Figure 1:**
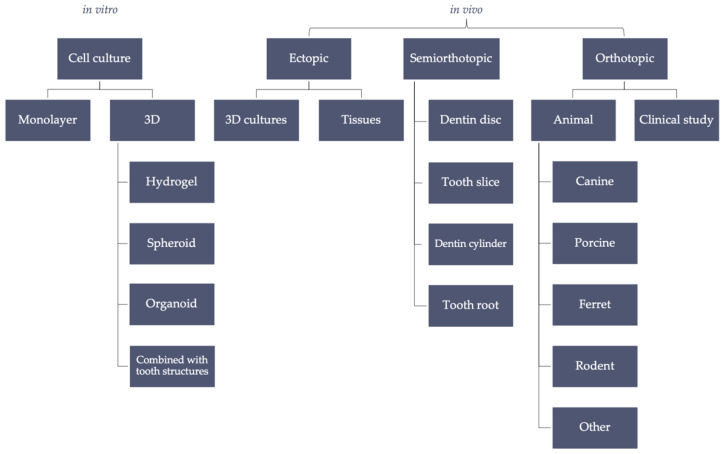
Compilation of study models for dental pulp tissue engineering.

The aim of this review is to compile different study models for both cell-based and primarily cell-free tissue engineering approaches for pulp regeneration that have emerged and developed over the past years. These can be grouped roughly into categories: in vitro; in vivo ectopic, referring to the ectopic transplantation of scaffolds and cells into immunocompromised animals; in vivo semiorthotopic, where cells are cultured in a tooth framework, which is transplanted into animals; and in vivo orthotopic, meaning the in situ simulation of clinical procedures in study animals, as shown in Figure 1. The advantages and disadvantages of the available in vitro and in vivo models are compared and discussed.

## 2. In Vitro

### 2.1. Monolayer Cell Culture

The monolayer cell culture presents the most basic laboratory technique. Distinguished by the locations of their origins, several cell sources have been identified. Of particular interest for dental pulp tissue engineering are mesenchymal stem cells, such as dental pulp stem cells (DPSCs), stem cells from the apical papilla, and periodontal ligament stem cells [11,12,13]. Dental stem cells can be obtained from human teeth, as well as from other species [14,15,16]. Furthermore, the use of non-oral stem cells for dental pulp regeneration, such as umbilical cord stem cells or amniotic epithelial stem cells, has also been investigated [17,18].

In terms of dental pulp, stem cells are isolated from pulp tissue by enzyme digestion or the outgrowth method and then cultured in medium supplemented with fetal bovine serum [19]. As adherent cells, they attach to the bottom of the culture vessel and form a confluent monolayer. In this culture environment, many cell characteristics, such as viability, population doubling, senescence, gene expression, or differentiability, and their responses to signaling molecules or biomaterials can be assessed.

The strengths of this model are controllable and reproduceable experimental conditions [20]. Different aspects of a complex in vivo system can be simplified and explored mechanistically in an in vitro culture setting [21], and costs are also very low compared to other models in use [20]. However, there are some disadvantages as well. Due to its simplicity, it has limitations when it comes to reproducing physiologic conditions. This includes tissue architecture, cell–cell communication, cellular movement, and cell–matrix interaction [22]. An adequate rendering of biological processes is, therefore, difficult, and the model may produce misleading and nonpredictive data [20,23].

Still, it has been instrumental in the characterization of tooth-derived stem cells [24] and remains the basis of most research even today. Many attempts have been made to culture and analyze odontoblast-like cells in vitro by the addition of signaling molecules to stem cells [25,26,27,28]. Signaling pathways in these cells have been investigated [29,30], gene expression patterns during cell differentiation have been revealed [31,32,33], and mineralization has been observed through alkaline phosphatase or alizarin red staining [17,34,35]. Since the cells at the interface with dentin are an integral part of the pulp–dentin complex, this model can also be adapted to study the behavior of DPSCs seeded directly onto the surface of dentin disks [19,36]. Furthermore, dentin matrix proteins, which are rich in growth factors that modulate cell differentiation, can be isolated from human dentin and supplemented in cell culture media to study the behavior of pulp cells [28,37,38].

### 2.2. Three-Dimensional (3D) Cell Culture

**Figure 2:**
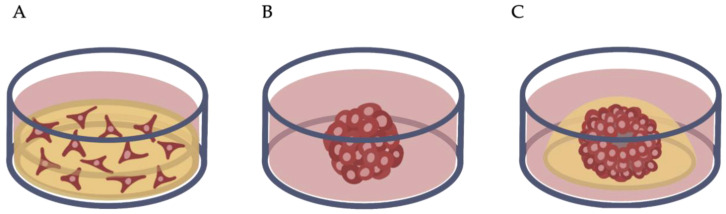
Three-dimensional cell culture models. (**A**) Hydrogel culture: cells are embedded in a scaffold material with a supernatant of culture medium. (**B**) Spheroid culture. (**C**) Organoid culture: cells are embedded in an extracellular matrix.

A two-dimensional approach can be enhanced by the utilization of three-dimensional culture methods, such as scaffold cell cultures, spheroids, or organoids (Figure 2). While scaffold cultures are mostly applied in material testing, spheroids and organoids were originally developed for tumor research and personalized medicine [23]. Unfortunately, the terms are used inconsistently in the literature. The term spheroid describes a conglomerate of adult cells without any scaffold, whereas an organoid consists of self-organized stem or progenitor cells forming organ-specific constructs with the help of a scaffolding environment [20,39].

**Figure 3:**
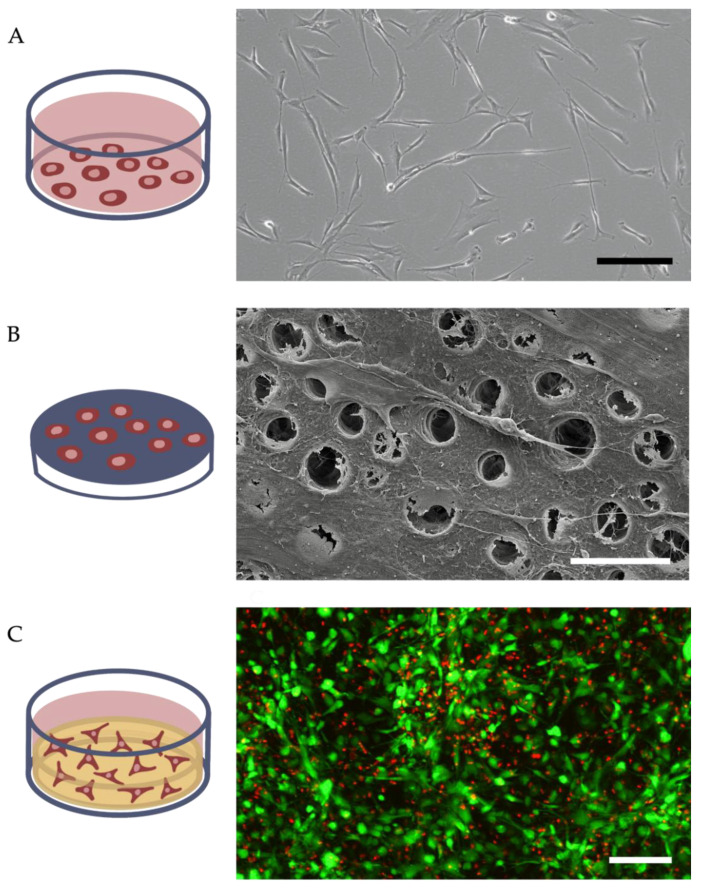
Comparison of morphologies of differently cultured cells. (**A**) DPSCs cultured on a tissue culture plate; scale bar of 200 µm. (**B**) REM image of DPSCs cultured on a dentin disk, where cells extend their processes into dentin tubules; scale bar of 10 µm. (**C**) Confocal laser scanning microscopy of live (green) and dead (red) DPSCs cultured in a collagen hydrogel shows the high turnover of cells; scale bar of 200 µm.

These three 3D-culturing methods have in common that the cells are spatially distributed within a supporting structure, i.e., an extracellular matrix [40,41,42]. 3D-cultured cells differ in morphology and physiology from two-dimensional cultures, as nonadherent cells are given the possibility to unfold their cellular shape and display greater heterogeneity, either in morphology, lineage, function, or age (Figure 3). Whereas necrotic cells in 2D cultures quickly detach from the surface of the culture flask and are rinsed out, 3D cultures consist of cells in different stages of aging. The core of agglomerates is often composed of necrotic cells, while the outermost layer consists of viable and proliferating cells, which emulates natural processes more closely [23]. The matrix itself also influences the cell behavior, e.g., by its rigidity. Stiff matrices can drive stem cell differentiation towards the osteogenic line [43].

In general, 3D cell cultures are more apt to reflect in vivo mechanisms than monolayer cultures [23] and, therefore, produce more accurate insights [44]. Thus, 3D cultures have the potential to bridge the gap between simple cell cultures and in vivo experiments, which can reduce the need for ethically challenging animal models [41]. However, as of now, 3D culturing is less established than monolayer culturing and is associated with greater effort and higher costs. Furthermore, the analysis of cell cultures is more difficult since cells need to be separated from the extracellular matrix, and high variability in the produced agglomerates reduces reproducibility [22,23].

Since there is always a close connection between hard and soft tissues in the pulp–dentin complex, it is also possible to combine 3D cell cultures with tooth structures. In this case, the pulp cavity of a slice of a tooth crown or the empty canal of a root can serve as reservoir to receive 3D-cultured cells. These constructs can be maintained in culture and studied in vitro [45,46]. Nevertheless, tooth slice or root fragment models are commonly used in in vivo model situations, which is discussed in detail in a following section.

#### 2.2.1. Hydrogels

Scaffolds can not only serve as a cell matrix in vitro, but constitute a pillar of tissue engineering, which makes them the subject of research in the realm of pulp regeneration. Essentially, these can be divided structurally into porous scaffolds, fibrous scaffolds, and hydrogels, where hydrogels are primarily used in the field of pulp biology research. They best imitate the mechanical properties of the dental pulp, and furthermore, their injectability makes them suitable for use in the root canal [47,48]. Appropriate materials should restore tissue architecture and guide cell growth but also degrade over time to provide space for new tissue formation [48,49]. The combination of scaffold materials and stem cells in vitro lays the foundation for assessing the eligibility of materials for clinical applications. Herein, cells can be cultured inside or on top of a hydrogel material to assess both the scaffold properties [50,51], such as inductivity or degradability, and the cell behavior, such as proliferation and migration, as well as cell–cell and cell–matrix interactions. Furthermore, the cytotoxicity of dental materials can be tested in a hydrogel model by adding substances to the culture medium or in direct contact with cells [52].

To emulate the mechanical and functional relationship between hard tissue and cells within the pulp–dentin complex, dentin can also be incorporated into three-dimensional culture systems. Rosa et al. filled tooth roots with stem cells from exfoliated deciduous teeth (SHED) that were encapsulated in a collagen matrix and cultured the fragments in vitro to investigate whether odontoblastic differentiation of cells was possible in full-length roots [46]. 

An innovative method for producing scaffolds and even hydrogels to incorporate cells is 3D bioprinting. Two different approaches are usually applied: one is the printing of acellular scaffolds, such as PCL [53], and the other is the additive manufacturing of scaffolds that already contain cells and signaling molecules [54,55]. Both types can be used in vitro and in vivo and are captivating because of their rapid fabrication, high precision, and customized production; however, the limitations of a low number of suitable materials, high costs, and possible undesirable additives could restrict applicability at present [56].

#### 2.2.2. Spheroids

Spheroids can be defined as a conglomerate of cells that self-assemble or are forced to aggregate [22,44]. They can be produced from a single cell type or as a multicellular spheroid and can be fabricated in a myriad of ways, from the gravity-enforced hanging drop method to the layering of cell sheets, as well as low-attachment culture plates [57], pellet culturing [58], the utilization of micro molds [59], and magnetic levitation [41].

In terms of pulp regeneration, researchers are studying the possibility to use in-vitro-generated cell agglomerates to replace damaged pulp tissue directly, but the use of engineered pulp tissue replicas also provides a novel model to study the processes in dental pulp regeneration and to assess the biocompatibility of various materials used [60].

#### 2.2.3. Organoids

Organoids commonly refer to self-organizing 3D cell structures of organ-specific cell types that arise from the differentiation of stem or progenitor cells [20]. They partially resemble the architecture and function of the target organ and are usually fabricated using decellularized extracellular matrices, such as Matrigel or collagen, to mimic a tissue’s noncellular components [61]. Matrigel is a commercially available material that contains structural proteins, such as collagen, elastin, and laminin, comparable to the basal lamina in vivo. However, it is not suited for in vivo application, as it is extracted from mouse sarcoma cells [62].

Organoids are, to a certain degree, able to simulate the architecture and functionality of a native organ [20,63]. Fashioned from embryonic cells, for example, organoids can recreate both hard and soft tissues. Cells in an organoid can be cultured for an extended time and mimic signaling pathways and niche conditions more closely compared to cells in a 2D system. Compared to an animal model, the implementation of organoids provides greater accessibility and feasibility [64]. However, the creation of organoids also requires certain laboratory skills, and protocols, including which cells and signaling molecules to use, still need to be revised [64].

Thus far, intestinal, cerebral, and renal organoids have been established [65]. Research into oral organoids is also being conducted, e.g., salivary glands have been recreated that can restore nerval connections and produce saliva when implanted orthotopically [66]. Jeong et al. managed to construct dentin-pulp-like organoids that expressed odontoblast-like markers and issued a biological response to the application of hydraulic calcium silicate cements [60]. Xu et al. also established an organoid model that was recommended for the toxicity screening of dental materials used, e.g., for direct pulp capping [67].

Outside the realm of dental pulp regeneration, researchers have even attempted to engineer whole tooth germ organoids. This has been partially successful by layering a multitude of different cell types [68,69]. These constructs display odontogenic markers and are also capable of epithelial invagination into the mesenchymal layer, mimicking the tissue interactions and signaling pathways at play during human tooth development [70]. Furthermore, the vision for these organoids is to replace dental implants, but further development is necessary [64].

#### 2.2.4. Bioreactors

One drawback of 3D cultures is that nutrients cannot efficiently penetrate the center of the 3D structures and waste accumulates, which affects cell survival. Consequently, these cultures are difficult to maintain for longer time periods [60]. However, in an in vivo environment, a steady blood supply guarantees tissue homeostasis. Bioreactors are, therefore, designed to mimic this natural phenomenon and to actively supply cells in the depth of 3D structures with nutrients and oxygen [71]. Examples for simple bioreactors are magnetic rod stirrers, rockers, rotating wall vessels, and peristaltic pumps [72,73,74]. What these methods have in common is that they set culture medium in motion in order to achieve deeper penetration into matrix structures. By choosing either laminar or a more turbulent flow, mechanical stimuli, such as sheer stress, flow-induced pressure, or dynamic compression, can also be applied to cells in culture vessels to further emulate an in vivo situation [71]. Naturally, each individual tissue requires specific stimuli. For example, cells differentiating towards an osteogenic cell fate have proven particularly perceptive to hydrostatic pressure and sheer stresses [75,76,77]. However, which stimuli best support the odontogenic differentiation of cells remains to be determined.

#### 2.2.5. Tooth-on-a-Chip Model

The so-called “organ-on-a-chip” techniques can be viewed as an extension of bioreactors. Here, cells are seeded in a microfluid device that ensures nutrient transportation through small channels and recreates physical parameters, such as pressure or shear stress [20]. Monitoring tools can also be included in this device [20]. The first organ to be emulated in a small plastic device was the lung. For example, the alveolar–capillary barrier was simulated by combining alveolar epithelial and endothelial cells, with both blood and oxygen flow, as well as cyclic mechanical stretching, in a 3D multichannel microfluid culture vessel [78]. França et al. were the first to build a tooth-on-a-chip model [79]. It consisted of two separate, closed-circuit channels filled with medium and two reaction chambers separated by a dentin fragment. Dental stem cells were added to one side, whereas the other side of the dentin barrier mimicked a tooth cavity. This model was used to test cell reactions to biomaterials by injecting solvents of the materials into the cavity side of the chip. Morphological changes in the cells could now be observed by direct cell imaging [79]. With further development, this approach holds many opportunities to enhance research into materials and signaling molecules used in dental pulp tissue engineering.

## 3. In Vivo Ectopic and Semiorthotopic Models

The transplantation of biological samples into the subcutaneous space of experimental animals is another method to create a physiological environment. In this context, ectopic means that tissues or cells are transplanted into experimental animals at a nonphysiological location. Cells in scaffolds can be transplanted by themselves or with signaling molecules [12,80,81,82,83]. However, especially in the context of pulp biology, cells are often implanted together on dentin disks [84,85], in tooth slices [59,86,87,88], in dentin cylinders, or in tooth roots [46,50,89,90,91,92,93] in order to simulate their natural environment. Since the directly surrounding or adjoining tissue is not ectopic, but rather corresponds to the natural environment (orthotopic), the term semiorthotopic is often used [94]. Here, the proximity to blood vessels enables nutrient supply to cells and the removal of waste products, and the animals can, thus, be considered in vivo bioreactors [71]. Additionally, interactions with resident peripheral nerve cells, connective tissues, and the immune system can be studied. Immunodeficient animals are most often utilized to prevent unwanted immunogenic reactions.

Implantation sites can vary. Small incisions through the skin can, for example, be made on the dorsum of mice, and subcutaneous pockets created by blunt dissection. After implant placement, wounds are closed by stapling or stitching [88]. Due to its abundant blood supply, the rat renal capsule is another location for ectopic transplantation; however, it is more difficult to access, and the mortality rate of experimental animals is higher than after subcutaneous implantation [95,96]. The subcutaneous implantation of autologous dental pulp cells or scaffold constructs into the dorsal surface of rabbits was also suggested as a valid ectopic model [97]. Ruangsawasdi et al. investigated the implantation of cell-free tooth roots filled with fibrin into the calvaria of rats and found that this placement produced more tissue ingrowth in the same time period than the dorsal location. This article suggested that rat calvaria could provide a microenvironment similar to the tooth socket [98].

Favorable outcomes can be achieved with ectopic and semiorthotopic transplantation, as they offer very translational features, are reproducible, and are well-described in the literature. Compared to other preclinical in vivo models, the utilization of smaller animals, such as mice, is preferred, as breeding and housing are less expensive and murine anatomy is well-understood. The surgical procedure of implant placement is easy to perform and results in minimal distress for the animals. Nevertheless, ethical concerns still need to be considered, and especially in the early stages of research, cell cultures should be preferred. The decision to use animals should never be taken lightly. It must also be noted that newly formed tissue, blood vessels, or nerve fibers can be of human or rodent origin. These ambiguities need to be kept in mind and reviewed in order to draw the correct conclusions regarding tissue formation (Table 1).

Further variations of these model are presented below, and selected references for applications of both in vitro and in vivo ectopic and semiorthotopic models can be found in Table 2.

### 3.1. Dentin Disk and Tooth Slice

**Figure 4:**
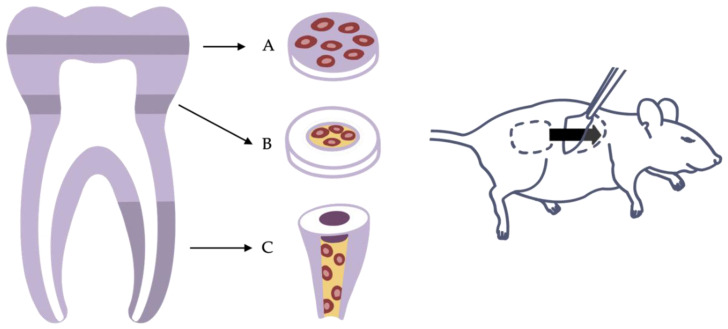
Variants of the ectopic transplantation model. (**A**) Dentin disk with cells seeded on top. (**B**) Tooth slice with cells and scaffold inserted into the pulp chamber. (**C**) Root fragment model with cells and scaffold inserted into the root canal.

Despite the fact that various research applications are based on the ectopic implantation of cells alone or cells encapsulated in a scaffold material [24,83,106], pulp cannot be restored without considering the pulp–dentin complex. The close mechanical and functional connections of cells and dentin are the reasons why many researchers choose to combine pulp-derived cells with dentin disks or tooth slices in vitro and implant them subcutaneously. Therefore, dentin disks or tooth slices are usually obtained in the area of solid coronal dentin or the pulp cavity from human molars respectively. The cells can then be seeded on top of solid dentin disks or cast within a scaffold into the former pulp chamber [45,87,88] (Figure 4A,B).

The tooth slice model has proven to be a valid semiorthotopic approach to observe and evaluate mechanisms of differentiation, vascularization, and regeneration [45,105]. It can answer questions regarding the pretreatment of dentin surfaces, the host integration of transplants, the deposition of extracellular matrix, and tumorigenic potential. Furthermore, the assessment of various scaffold materials and their suitability for regenerative procedures is possible [107]. The research team around Nör has used it to investigate genetically modified DPSCs and to better understand signaling pathways [29,30]. It also allows for the transplantation of traceable cells to analyze cell fate in vivo [86,105].

### 3.2. Dentin Cylinder and Tooth Root

Sufficient vascularization is a prerequisite for cells to survive and generate new tissue [71,86,108]. In a tooth slice model, nutrients and oxygen may reach the cells easily by diffusion from neighboring tissues, as the diffusion distance is short. However, the anatomy of an actual tooth is different. Diffusion from the root tip all the way to the crown is not possible. Only the advancement of a functional vascular system allows cells to expand into the entire pulp cavity and tissue to develop even far from the apical entry [108,109]. As blood vessels have only restricted access to the root canal through the apical foramen, models using dentin cylinders or tooth roots mimic the difficulties of the clinical situation more accurately. Here, whole roots or parts of them are separated from extracted teeth, prepared, and filled with cells and a scaffold material. Sample constructs can then be implanted, for example, into a mouse dorsum to be accessed by blood vessels and nerve fibers (Figure 4C). Whereas leaving both ends of the dentin cylinder open may provide optimal blood supply from two directions, sealing of the coronal opening with a bioactive material corresponds to clinical situations, as the unilateral sprouting of vessels into the tooth root presents a challenge [46,91,92]. However, the decision of how to prepare the roots must be made depending on the application and the specific research question.

**Figure 5:**
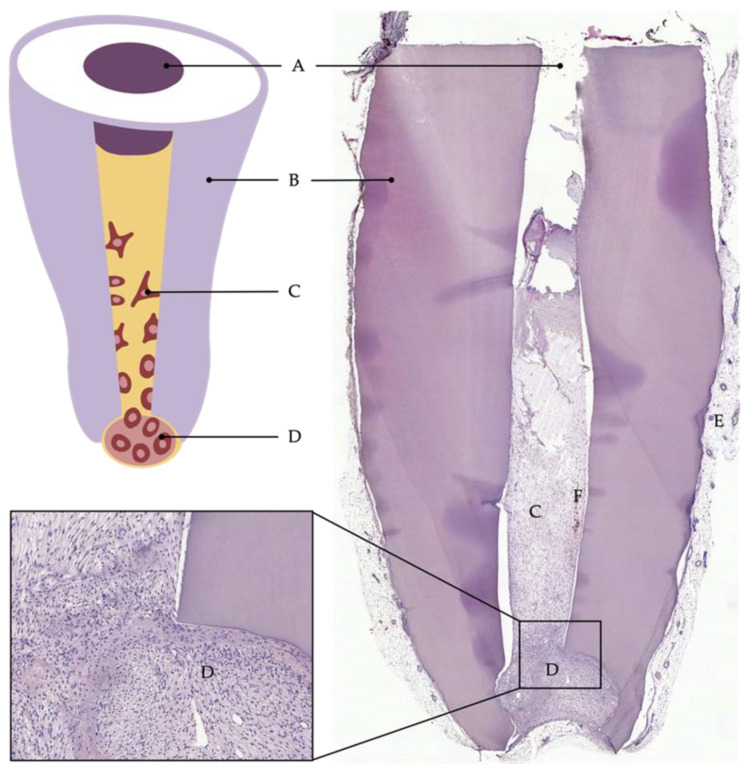
Cell-homing model. Tooth root recovered after 6 weeks of implantation into subcutaneous dorsal space of mice: (**A**) coronal plug, (**B**) dentin of root walls, (**C**) cells that migrated into the root canal, (**D**) apical reservoir of stem cells in collagen, (**E**) murine tissue, (**F**) blood vessel.

This semiorthotopic model situation allows a variety of investigations and analyses. The focus can be on qualitative factors, such as the formation of odontoblast-like cells or the expressions of certain markers, as well as quantitative factors, such as the number of blood vessels or nerve fibers or the amount of newly formed tissue. Furthermore, the model has been continuously developed and modified over the past years to answer specific questions or to counteract limitations. For example, Widbiller et al. established a customized tooth root model to test cell-homing approaches for dental pulp regeneration [91]. Here, the root canal was filled with a growth-factor-laden hydrogel with the ability to promote chemotaxis. Stem cells were then placed only at the apical opening of the root to mimic the apical papilla as the stem cell source of immature teeth. After the recovery of the tooth roots from the mouse subcutaneous space, the samples can be processed histologically, and the newly formed tissue can be analyzed by various techniques (Figure 5) [110].

Another interesting variant was reported by Hilkens et al. with the aim of creating a more standardized situation. Cells were seeded not into human root fragments but into 3D-printed hydroxyapatite scaffolds shaped as tooth roots that were then implanted into mice to assess the angiogenetic potentials of different stem cells [109].

## 4. In Vivo Orthotopic Model

**Figure 6:**
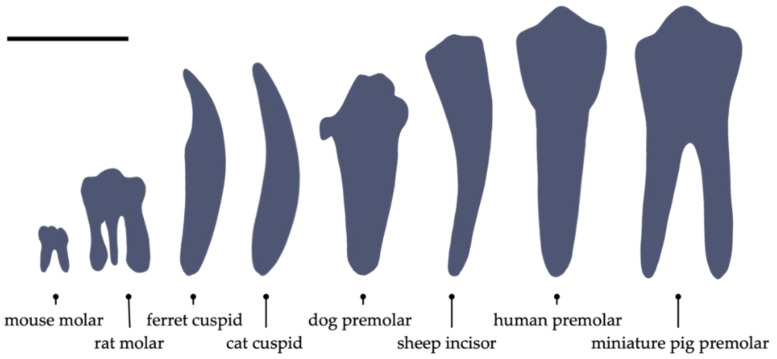
Relative tooth sizes of different species. Scale bar: 1 cm.

Lastly, the most translational situation to investigate stem pulp tissue engineering is the experimental animal. In this orthotopic model, signaling molecules, a scaffold, and eventually, stem cells are implanted together into an anatomically correct site, which is the root canal of a tooth in its physiological position in the oral cavity of an animal. Animal models for dental pulp tissue engineering can be grouped into small animal models and large animal models. Large animal models, such as dogs and pigs, are often preferred because it is easier to facilitate from a treatment perspective regarding tooth size (Figure 6) [95].

The scientific value, thereby, is that treated teeth can be examined histologically after animals have been euthanized, and the treatment outcomes of regenerative procedures can be systematically evaluated at tissue and single-cell levels. Furthermore, orthotopic models are used to evaluate the efficacy and quality of proposed regenerative strategies and to establish a data basis to design future clinical trials adequately [95]. The fields of application cover many areas, such as tumorigenesis, the testing of restorative materials, and root canal disinfection methods, as well as periodontal and endodontic regeneration [93,111].

However, it must always be kept in mind that conclusions derived from animal studies are not necessarily transferable to the clinical situation. Tooth anatomy, as well as the local microbiomes or regenerative capacities of cells or tissues, may differ. For example, autologous stem cells of animals, which are typically applied to circumvent the problem of immunocompatibility, may not behave the same way as human stem cells [111]. Furthermore, animal studies are afflicted with higher expenses than in vitro methods, which limits their availability and feasibility [111]. Most importantly, ethical concerns must always be considered when conducting research in vivo, and the step from cell culturing to animal testing should not be taken carelessly. From an ethical point of view, every single sacrifice of an animal needs to be justified by an increase in scientific knowledge. Therefore, the proposed research protocol must be reviewed before experiments can be initiated. It must align with local animal welfare laws and regulations, and it is important to ensure that researchers are educated in the handling of the animal in use. In addition, the least sentient animal should be preferred when choosing a suitable species (Table 3) [71]. When planning animal-based research, one should consider the principle of the three Rs: (1) replacement with alternative methods, such as in vitro cell cultures, whenever possible; (2) reduction in number, which may include performing multiple experiments on the same animal; and (3) refinement of the projects and techniques used in order to minimize pain and stress [112,113]. However, a reduction in number that results in invalid data and the need for repetition of the experiment is to be avoided [71]. When reporting the findings of animal studies, the ARRIVE guidelines (Animal Research: Reporting of in vivo Experiments) should be observed [114].

### 4.1. Large Animal Models

#### 4.1.1. Dogs

Dogs have often been used as a model in dental pulp regeneration research [115]. In general, canine teeth are similar to human teeth in anatomy, growth patterns, and pathophysiology [116]. Canine premolars are preferred, as they present the greatest similarity to human molars [71]. However, even other teeth, such as incisors or canines, are suitable [117].

Differently from human teeth, the root canal system of an adult dog ends in a highly branched apical delta with multiple ramifications, which makes disinfection by irrigation difficult [116]. The premolars of younger dogs, however, have not yet formed the complex delta and still present a more stringent apical anatomy [71]. Still, the enlargement of the apical opening is a necessary step during the operation procedure [118]. Of course, there are ethical concerns and public criticism associated with the use of the canine model, which are justified and understandable, as dogs are considered companions to humans and are usually kept as pets.

Overall, there is extensive knowledge of this study model and, thus, its predictability in outcome can be seen as an advantage. Groups around Nakashima and Iohara have established and refined canine models using the beagle dog breed due to its friendly temperament and small size [118,119], which is advantageous for the housing and handling of the animals. They have worked for many years on the development of clinically applicable protocols for pulp regeneration by cell transplantation, making extensive use of the canine model, thereby, for example, proving the successful regeneration of pulp tissue using cell transplantation approaches by transplanting autologous canine stem cells into canine teeth after partial or total removal of the pulp [108,118]. Furthermore, this experimental set-up has been used to investigate different subclasses of canine dental stem cells [120], the influence of various signaling molecules [58,120], and the impacts of age [121] and inflammation [122] on endodontic regeneration.

#### 4.1.2. Pigs

Pigs are used in various areas of research, especially as surgical models. This is because their growth patterns, physiology, and head size come close to humans [123,124]. Endodontic procedures were performed on porcine premolars, which were easy to access and were deemed suitable for experimentation in dental pulp tissue engineering [93]. Another advantage is that euthanizing pigs, which are regarded as livestock, is considered less critically [71,117]. However, there are also shortcomings to this model, such as the heavy weight of the animals compared to dogs, the small size of pig pulp chambers, and the challenges of adequate housing and high demands for feeding and care [93]. Additionally, their posterior teeth were described as difficult to access, and the root canal morphology was irregular and not ideal [93]. Interestingly, pigs were reported to possess a “disobedient temperament” or “uncooperative behavior”, which deemed them difficult to manage [117]. However, Zhu et al. isolated porcine dental pulp stem cells and could prove the formation of vascularized pulp-like tissue in pig teeth and reparative dentin formation [93]. On the other hand, the implantation of porcine dental pulp stem cells in induced pulp defects did not result in regenerated pulp or reparative dentinogenesis in other studies [124]. Therefore, there are still challenges that need to be overcome before this model can find widespread use.

### 4.2. Small Animal Models

#### 4.2.1. Rodents

Small animal models are often automatically excluded from use as orthotopic study models because of the diminutive size of the teeth. In addition, rodent incisors grow continuously throughout their lives and are only shortened due to attritive wear and tear. In contrast, their molars are brachydont and can, therefore, be considered for endodontic treatments. However, small mouth size limits access, and teeth are minute compared to human teeth. When using standard endodontic instruments, there is a high risk of perforating the soft dentin walls, especially in curved roots. Nowadays, the use of magnification by, e.g., operative microscopes and small instruments enables the use of rodents for endodontic applications [71,125,126]. Despite the difficulties in treating teeth, the animals’ small sizes are beneficial when it comes to housing. Another advantage is that rodents possess faster biological responses to treatment; one month for rats is equal to 30 months in humans [125].

Thus, the rat model was reported as a suitable model to study novel methods of root canal treatment after apical periodontitis [126]. On various occasions, rodent models have also been used for the study of pulpal healing in direct pulp capping [127,128,129]. Furthermore, Almushayt et al. used rats to test the functionality of DMP1 as a signaling molecule for dental pulp tissue engineering [130].

#### 4.2.2. Ferrets

The ferret is a medium sized carnivore that is much smaller than dogs or pigs. Their teeth exhibit anatomical, physiologic, histologic, and pathologic characteristics that resemble human teeth [111]. In particular, their single-rooted canine is suited for endodontic procedures [71,131,132].

Ferrets have the advantage of being less expensive to house and easier to maintain and breed in the laboratory than larger animals and are typically not considered as pets [111]. Because their root apices are wide open, ferret teeth lend themselves to the study of regenerative endodontic procedures where the pulp tissue is removed and bleeding is induced in order to facilitate the formation of new tissue in the root canal [133,134]. In addition, periapical infections can predictably be induced, and ferret canines can be used to investigate irrigation and medication protocols [131].

### 4.3. Untypical or Inappropriate Models

#### 4.3.1. Feline Model

Other animals have been considered for stem-cell-based oral tissue engineering, as well. Cats are easy to anesthetize and have four single-rooted cuspid teeth that are suitable for endodontic procedures. However, they are more expensive to accommodate than small animals, and in analogy to dogs, they are commonly considered as pets, which induces emotional problems and public objections [111]. Although they have been described historically as a possible model, e.g., for the study of periapical lesions [135], they have not been used as such for a long time.

#### 4.3.2. Ovine Model

Sheep present a less-developed study model in dental research but were reported to be very promising [117]. Because they are ruminants, the salivary pH of sheep is higher than humans [117]. Furthermore, ovine teeth are different from those of humans, although there are similarities in anatomy and size [136,137]. The permanent first incisors of 12-to-18-month sheep are suited for regenerative endodontic studies, as they possess an open apex and thin dentinal walls. Further advantages can be seen in the low ethical concerns regarding farm animals and the easy upkeep, as they can be released on fields [137]. Even if sheep have been used in other research areas, such as periodontology [138] or bone regeneration [139], further investigations need to be conducted before sheep can be utilized as a study model for dental pulp tissue engineering [136].

#### 4.3.3. Primate Model

Because of their sentient character, long life span, and expensive acquisition and care, non-human primates are not an adequate model for research in dental regeneration [140,141]. Furthermore, despite presenting great anatomical similarities to humans, non-human primates are not ideal for endodontic research, as they have far better recovering abilities than humans. The artificial induction of pulpitis was hindered by the strong resistance of primate pulp to oral contamination [71]. For various ethical, legal, and physiological reasons, primates may not be used in this context, and other animal models must be preferred.

## 5. Conclusions

Today, various 3D cell culture models offer good alternatives to animal studies. Certain questions can easily be resolved in vitro, and the ongoing development of organoid and spheroid cultures, for example, could expand this area of application in the future. In order to gain further insight into outcomes in a physiological environment, there is, of course, also a necessity for animal studies. In consideration of the 3 Rs, study designs based on the semiorthotopic approach are of great benefit here. However, the final investigation of the research goal must be carried out in an in situ approach. Small animal studies should also be considered in this context in order to reduce the number of currently used large and more sentient animals.

Looking at the variety of in vitro and in vivo study models, there is not a single model that is suitable to answer all questions related to dental pulp regeneration. In each case, the appropriate model situation must be selected to correspond with the specific research question and the current state of development on the way to clinical application. Requirements, costs, and above all, ethical considerations should be included in the decision-making process.

## Figures and Tables

**Table 1:** Strengths and weaknesses of in vitro and in vivo models.

	In Vitro		In Vivo	
	Monolayer	3D Culture	Ectopic	Semiorthotopic
high cost	+	+	++	++
ethical concerns	+	+	+++	+++
literature experience	+++	+	++	++
difficult implementation	+	++	++	++
reproducibility	+++	++	+	+
mimicry of natural situation	+	++	++	+++

**Table 2:** Selected references for applications of each study model, including both in vitro and in vivo. The asterisk indicates categories that are not conceivable in the present classification.

Study Models	In Vitro	In Vivo	
		Ectopic	Semiorthotopic
Scaffold culture	Wang et al., 2010 [81] Galler et al., 2012 [50] Qu and Liu, 2013 [40] Widbiller et al., 2016 [52] Lin et al., 2021 [42]	Buurma et al., 1999 [80] Gronthos et al., 2000 [12] Wang et al., 2010 [81] Lee et al., 2011 [82] De Almeidas et al., 2014 [83]	*
Spheroid and organoid	Xiao and Tsutsui, 2013 [99] Dissanayaka et al., 2014 [59] Jeong et al., 2020 [60] Zheng et al., 2021 [100] Chan et al., 2021 [101]		*
Dentin disk	Sloan et al., 1998 [102] Huang et al., 2006 [19] Widbiller et al., 2019 [103] Atesci et al., 2020 [104]	*	Batouli et al., 2003 [84] Goncalves et al., 2007 [85]
Tooth slice	Casagrande et al., 2010 [45]	*	Cordeiro et al., 2008 [86] Prescott et al., 2009 [87] Sakai et al., 2010 [105] Casagrande et al., 2010 [45] Sakai et al., 2011 [88] Dissanayaka et al., 2014 [59]
Dentin cylinder and tooth root	Rosa et al., 2013 [46]	*	Galler et al., 2011 [89] Galler et al., 2012 [50] Rosa et al., 2013 [46] Takeuchi et al., 2015 [90] Widbiller et al., 2018 [91] With coronal plug: Huang et al., 2010 [92] Zhu et al., 2018 [93]

**Table 3:** Strengths and weaknesses of in vivo orthotopic models.

	In Vivo Orthotopic			
	Dog	Pig	Ferret	Rodent
high cost	+++	+++	++	+
ethical concerns	+++	++	++	++
literature experience	+++	++	+	+
housing requirements	++	+++	+	+
animal handling	+++	+	++	+++
similarity of tooth anatomy	++	++	+	+
similarity of tooth size	+++	+++	++	+
access to teeth	++	++	++	+

## Data Availability

Not applicable.
